# Unilateral axillary adenopathy induced by COVID-19 vaccine: US follow-up evaluation

**DOI:** 10.1007/s00330-021-08309-7

**Published:** 2021-10-16

**Authors:** Alba Cristina Igual-Rouilleault, Ignacio Soriano, Paola Leonor Quan, Alejandro Fernández-Montero, Arlette Elizalde, Luis Pina

**Affiliations:** Present Address: Clínica Universidad de Navarra, Avenida Pío XII 36, Pamplona, Spain

**Keywords:** COVID-19, mRNA vaccine, Unilateral axillary lymphadenopathy, Ultrasound

## Abstract

**Objectives:**

This study was conducted in order to investigate COVID-19 vaccine influence on unilateral axillary lymph nodes, comparing nodal basal features with their characteristics after the first and second vaccination dose.

**Methods:**

Ninety-one volunteer employees from our center who participated in the BNT162b2 (Pfizer-BioNTech) vaccination campaign were prospectively recruited. A total of three axillary ultrasound evaluations of the ipsilateral vaccinated arm were performed: before vaccination, the week after the first dose and the week after the second dose. The following findings were recorded: the total number of visible nodes, the maximum measurements of the diameter and cortex, Bedi’s classification, and color Doppler evaluation. The collected data were compared using paired-sample Student’s *t*-test for quantitative continuous variables and Wilcoxon rank-sum test for ordinal variables. Additional analyses were performed after classifying patients according to the previous history of COVID-19 disease. Differences among both groups were evaluated with the Mann–Whitney U test. Variables with a *p* value < 0.05 were considered statistically significant.

**Results:**

Comparative analyses between the three US examinations showed a statistically significant augmentation of total visible nodes, maximum diameter, cortical thickness, grade of Bedi’s classification, and Doppler signal (*p* < 0.001). Analyses between patients with and without previous COVID-19 infection showed a higher lymph node response in naïve patients compared to those who were previously infected.

**Conclusions:**

According to our results, both doses of COVID-19 vaccine induced an increase of all axillary lymph node parameters with statistically significant differences, especially in coronavirus-naïve patients.

**Key Points:**

*• Pfizer COVID-19 vaccine induces a high incidence of ipsilateral axillary lymphadenopathy.*

*• US scan identified an increase of all lymph nodes parameters, especially in coronavirus-naïve patients.*

## Introduction

A novel coronavirus, designated as COVID-19, 2019-nCoV, or SARS-CoV-2, suddenly emerged in December 2019 in Wuhan, China. The outbreak quickly spread around the world and 3 months later, the World Health Organization Global declared COVID-19 a global pandemic. Since then, about 181 million cases and 3.9 million deaths have been confirmed [1]. To take action on this public health emergency, individual and community-based measures, including social distancing and hygiene measures, have been implemented by international governments to mitigate pandemic fatalities.

Recently, an emergent vaccination program has been approved through the US Food and Drug Administration’s (FDA’s) authorizing novel mRNA (Pfizer-BioNTech, Moderna) and viral vector–based (AstraZeneca, Janssen) vaccines. The scientific community has already documented the strong immune response evoked by both types of COVID-19 vaccinations with predominantly local side effects as pain, tenderness, or swelling at the site of vaccine administration, with frequent concomitant development of reactive unilateral axillary lymphadenopathy [2].

Therefore, radiologists must consider recent COVID-19 vaccination history as a possible differential diagnosis for patients with unilateral axillary adenopathy. The preferred imaging technique for their assessment is ultrasound [3] and malignancy should be ruled out, especially during breast cancer screening, in the evaluation of symptomatic patients and in the context of recently diagnosed breast cancer.

Due to the lack of information about this newly diagnostic challenge and the current implementation of COVID-19 vaccination programs for the general population, the aim of our research was to assess the effect of the Pfizer-BioNTech vaccination on unilateral axillary lymph nodes, comparing nodal basal features with their characteristics after the first and second vaccination.

## Materials and methods

### Study design

Between February and April 2021, 171 employees from our center, with no history of previous cancer, were invited to participate in this prospective study. A total of 91 volunteers were finally recruited with the approval of our institutional review board and the written informed consent of all participants.

Volunteers selected were Pfizer-BioNTech COVID-19 vaccine recipients, requiring two injections given 3 weeks apart. The administration of both doses was performed in the same arm in all volunteers (preferably in the non-dominant arm) who underwent a total of three axillary ultrasound (US) evaluations of the ipsilateral vaccinated arm. The first axillary US exam was obtained within the week before vaccine administration (baseline); the second one, the week after the first dose (first follow-up); and the last one, the week after the second dose (second follow-up). The mean duration for the follow-up period was 32 days, starting from the baseline US examination and ending at the second follow-up after the last dose administration. Demographic factors—age and sex—were registered and all participants were asked retrospectively about prior history of COVID-19 infection.

Additionally, lymphadenopathy reaction outcome was analyzed offering a prospectively US follow-up examination to patients who showed a cortical thickness greater than 3 mm after the second dose. Patients’ follow-up period finished when their cortical thickness achieved normal values (≤ 3 mm). Follow-up study until regression to basal values was not performed.

### Image acquisition and assessment

Four radiologists, including two third-year residents and two radiologists with more than 20 years of experience in breast imaging, prospectively performed axillary US examinations of the vaccinated arm using two different broad-band linear transducers with a band frequency of 8–13 MHz (Logic E9, GE Healthcare, and Aplio i800 series ultrasound system, Canon Medical Systems Corporation). To reduce interobserver variability, each patient’s images were reviewed in consensus by the two expert radiologists.

Nodal findings recorded in our study included the total number of visible nodes, maximum measurements of the long-axis size and the cortical thickness, morphological Bedi’s classification, and color Doppler evaluation. The morphological evaluation of lymph nodes was conducted following the cortical Bedi classification, as follows: type 1, hyperechoic, no visible cortex or less than 1 mm; type 2, thin (< 3 mm) hypoechoic cortex; type 3, hypoechoic cortex thicker than 3 mm; type 4, generalized lobulated hypoechoic cortex; type 5, focal hypoechoic cortical lobulation; type 6, totally hypoechoic node with no hilum [4]. Finally, Doppler evaluation was performed using a four-degree scale routinely employed in our institution: degree 0, no Doppler signal; degree 1, only hilar Doppler signal; degree 2, mild-moderate positive Doppler signal in hilar and cortical regions; degree 3, high positive Doppler signal in hilar and cortical regions. All these features were registered choosing the maximum values of each variable, selecting different nodes if necessary (i.e., the cortical thickness and Bedi’s classification could be measured in one node, while the larger diameter could be measured from a different one).

### Statistical analysis

Data collection was recorded in an EXCEL database (Microsoft). The outcome measures of each variable were reported at three consecutive US exams: baseline, after the first dose and following the second one.

IBM SPSS Statistics software version 21.0 was employed to perform statistical analysis. Evaluation of the progressive values of data collected was studied using two comparative analyses: baseline examination versus the first follow-up and the first follow-up versus the second one. Paired-sample Student’s *t*-test and Wilcoxon rank-sum test were used to study quantitative continuous variables (number of nodes, long-axis size, and cortical thickness) and ordinal variables (Bedi’s classification and Doppler scale), respectively. Additional analyses were performed after classifying patients according to previous history of COVID-19 disease. Differences among both groups were evaluated with the Mann–Whitney *U* test. Finally, analysis of variance (ANOVA) test was used as a quality control to study disparity between the four radiologists comparing cortical thickness values in the second follow-up examination. Variables with a *p* value < 0.05 were considered statistically significant.

## Results

**Fig. 1:**
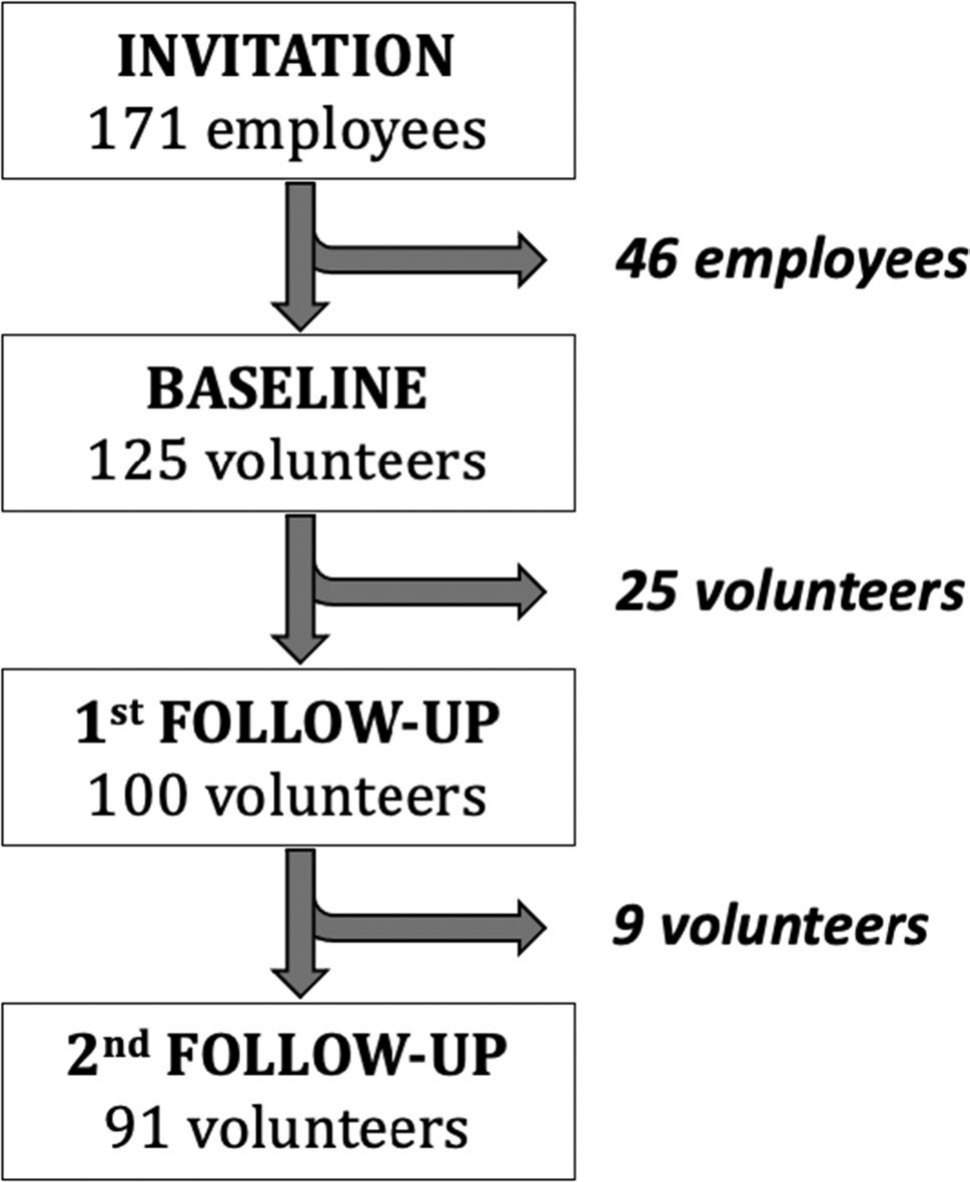
Flow diagram of the study selection process

The present study was presented to 171 employees of our center who participated in the Pfizer-BioNTech vaccination campaign. A total of 125 volunteers were recruited at baseline examination. However, during the follow-up period, 25 volunteers were absent from the US control after the first dose and 9 patients did not attend the last US exam following the second dose. A total of 91 patients were definitively enrolled in the ongoing study, resulting in a retention rate of 72.8% (Fig. 1).

Recruited patients were between the ages of 20 and 65, with a mean age of 44 years. The majority of included patients were females (approximately 80%) and previous history of COVID-19 infection was registered in almost 26 patients (28%) (Table 1).

**Table 1:** Details of sample

Age (years; range)	43.76 (20–65)
Women (percentage)	72 (79.1%)
COVID-19 (percentage)	26 (28.6%)

**Fig. 2:**
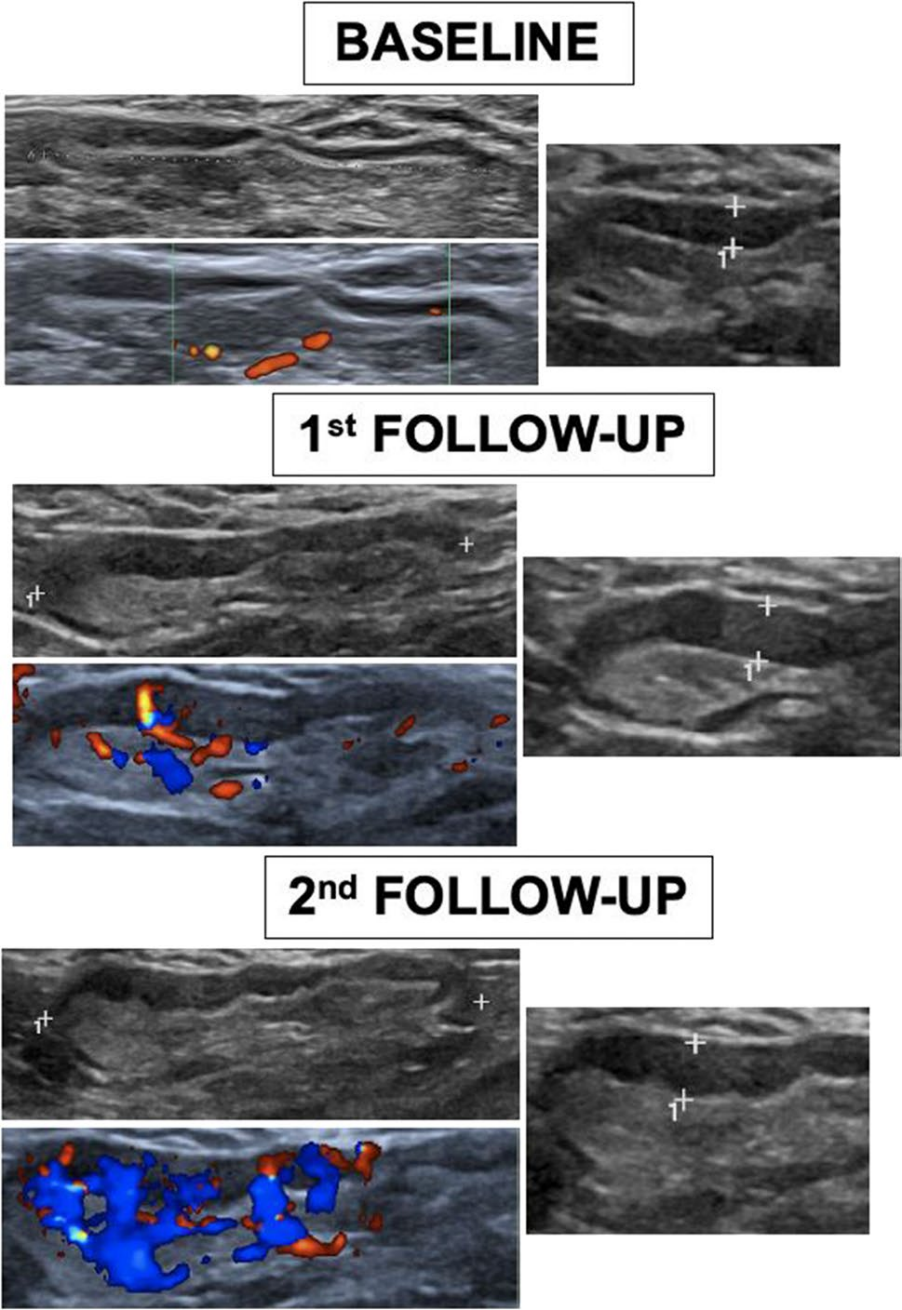
Comparative US images from one volunteer between baseline and the first and second follow-ups showing a significant gradual increment of maximum diameter, cortex, Bedi’s classification, and Doppler scale degree

**Fig. 3:**
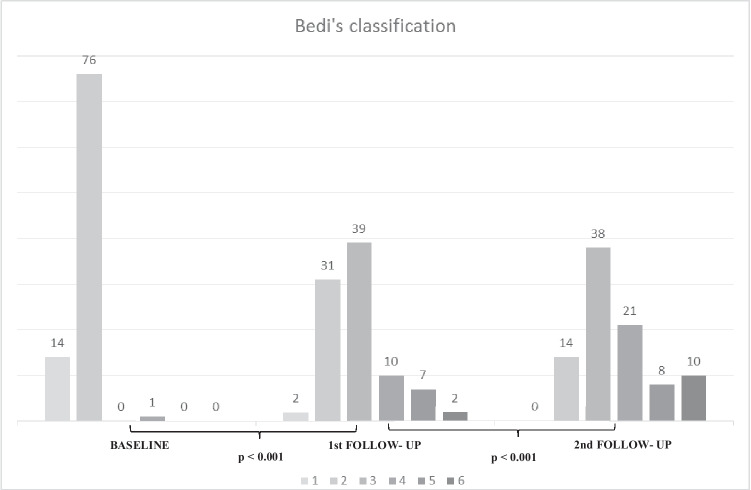
Sample distribution and comparison per Bedi’s classification grades in the follow-up period (number of patients indicated for each grade)

**Fig. 4:**
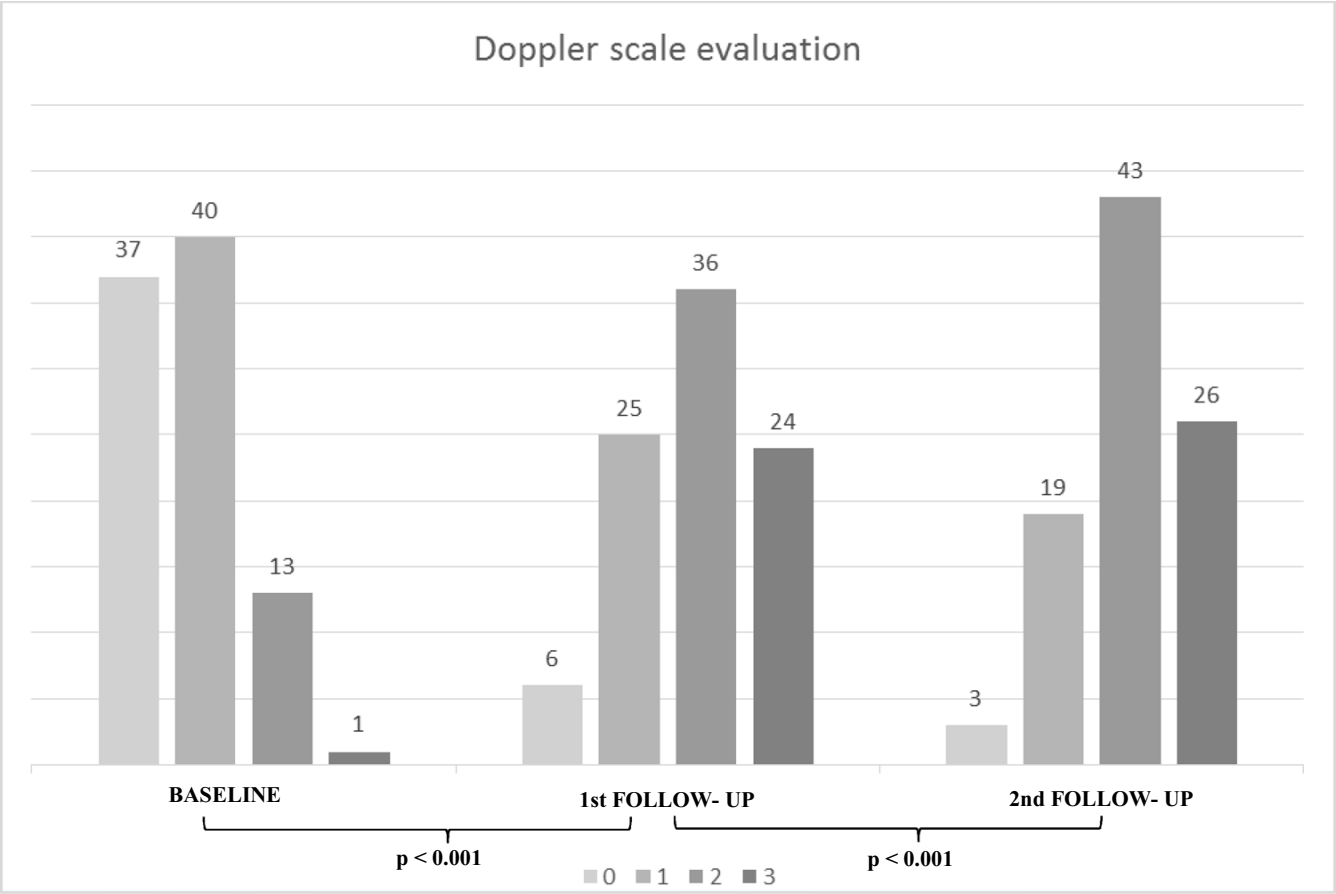
Sample distribution and comparison per Doppler scale degrees in the three consecutive controls

Global comparative analyses between the basal and the first US follow-up as well as the first and second US examinations showed a statistically significant gradual increment of all variables (Table 2), including quantitative and ordinal variables (Fig. 2). Regarding quantitative variables, it should be noted that a progressive increase of total visible nodes was observed, with 2.96 mean nodes at baseline, 4.77 mean nodes in the first follow-up, and 6.21 mean nodes in the second one (Table 3). Moreover, cortical thickness augmented from 1.6 mean mm at baseline to 3.7 mm in the first control and to 4.6 mm in the last control. In addition, statistical analysis of ordinal variables (grade of Bedi’s classification and color Doppler scale) also showed significant differences with a *p* value of < 0.001. Specifically, the comparison of sample percentage with a suspicious Bedi’s classification grade (types 3, 4, 5, and 6) between the three US controls evidenced the following: 1% at baseline vs 64% at the first US control and 85% at the last scan control (Fig. 3). Concerning Doppler signal evaluation, the most frequent degree detected was grade 1 at baseline with 44% of total percentage vs grade 2 at the first and second follow-ups with 40% and 47%, respectively (Fig. 4). After analyzing all the collected data, only one patient (26-year-old female with previous history of COVID infection) showed no significant changes during the full study.

**Table 2:**
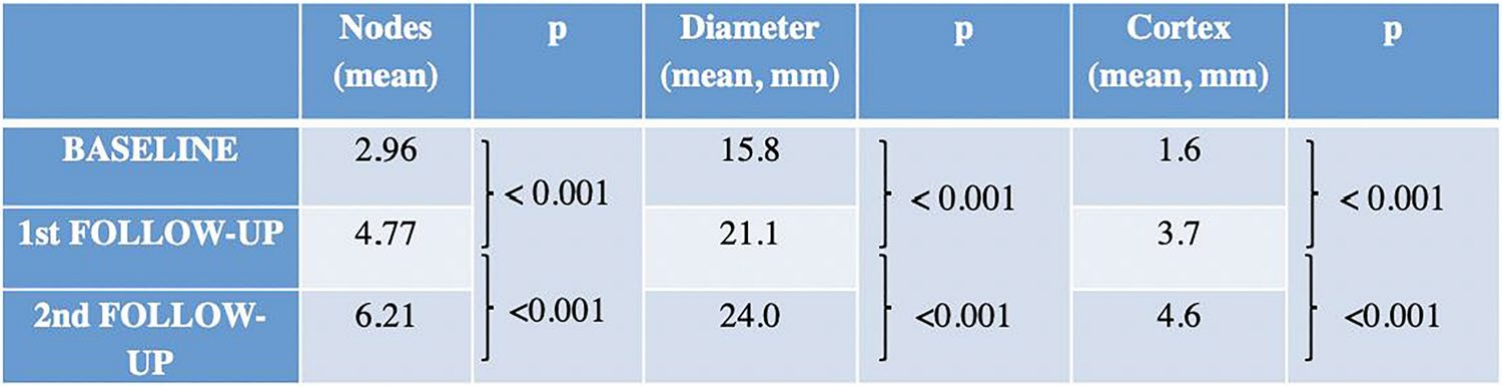
Mean values of quantitative continuous variables in the three consecutive US examinations

**Table 3:** Mean values comparison of quantitative continuous variables between two patient groups: patients who had experienced infection by SARS-CoV2 and patients who had not (mean ± SD)

	**COVID**	**NO-COVID**	
	Nodes (number)		*p*
Baseline (mean ± SD)	3.04 ± 1.12	2.92 ± 1.04	0.626
1^st^ follow-up (mean ± SD)	4.65 ± 1.62	4.82 ± 1.68	0.643
2^nd^ follow-up (mean ± SD)	5.23 ± 1.86	6.60 ± 2.51	0.006
	Cortex (mm)		*p*
Baseline (mean ± SD)	1.82 ± 1.33	1.48 ± 0.59	0.226
1^st^ follow-up (mean ± SD)	2.89 ± 2.14	3.99 ± 1.72	< 0.001
2^nd^ follow-up (mean ± SD)	3.49 ± 1.76	4.98 ± 1.85	0.001
	Diameter (mm)		*p*
Baseline (mean ± SD)	17.10 ± 1.37	15.33 ± 0.84	0.266
1^st^ follow-up (mean ± SD)	21.92 ± 1.40	20.82 ± 0.81	0.479
2^nd^ follow-up (mean ± SD)	23.20 ± 1.34	24.36 ± 0.82	0.462

**Fig. 5:**
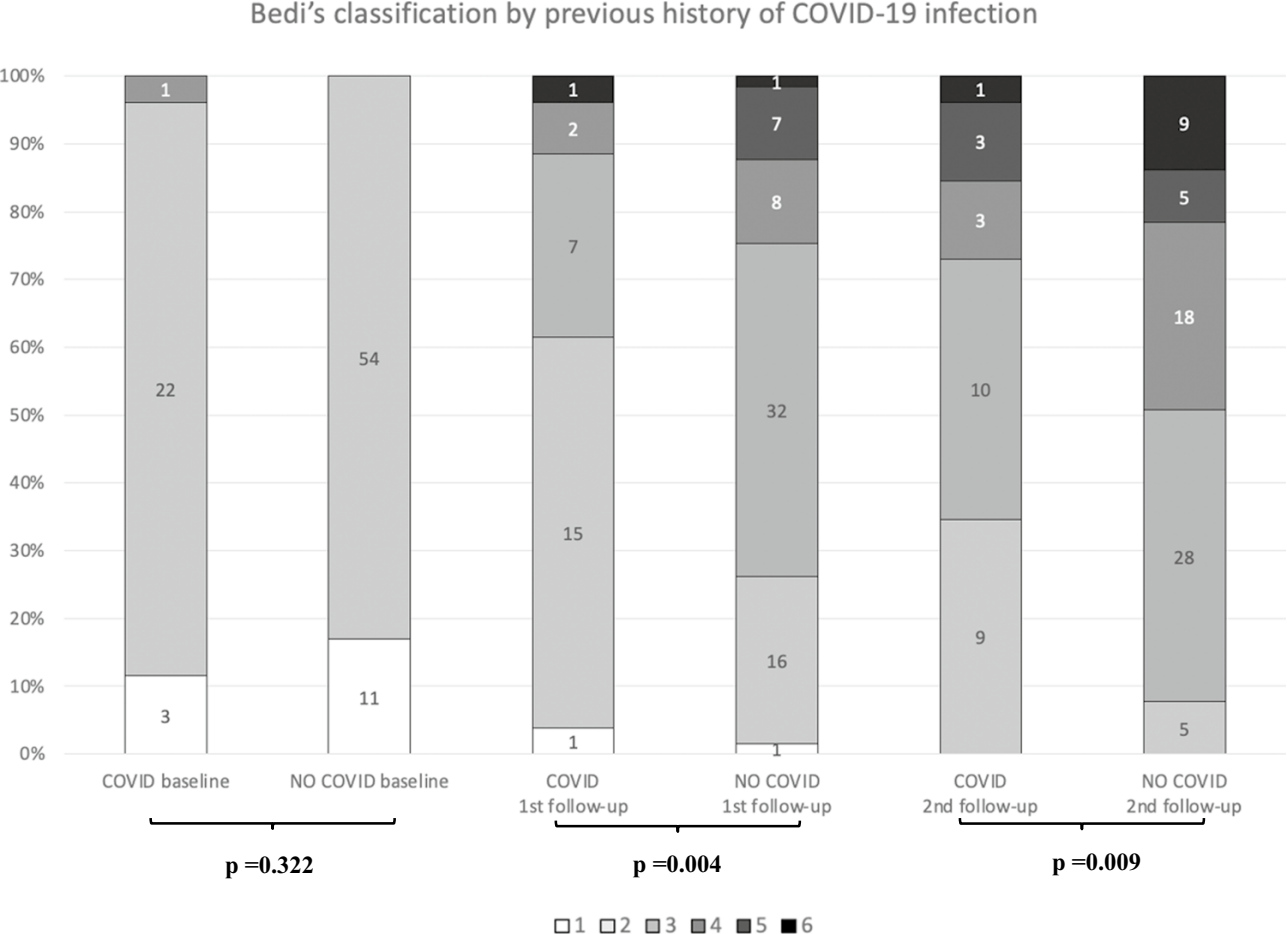
Sample distribution of both groups and comparison per Bedi’s classification grades in the follow-up period

**Fig. 6:**
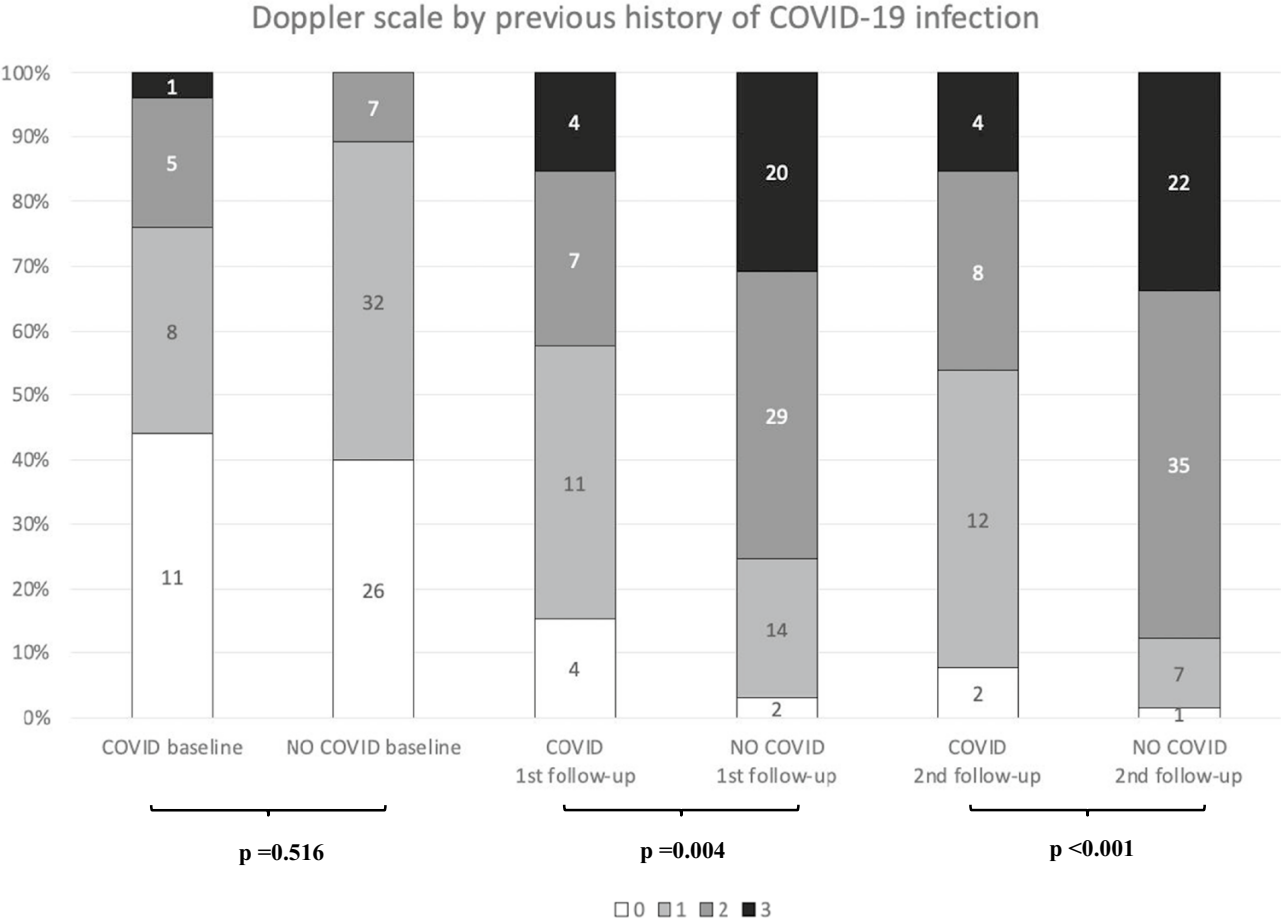
Sample distribution of both groups and comparison per Doppler scale signal in the follow-up period

Further analysis of axillary lymph node reaction induced by COVID-19 vaccine in patients with and without previous COVID-19 infection showed a more significant lymph node response in naïve patients compared to those who were previously infected by SARS-CoV-2. No statistical differences were found in any variables at basal US examination between both groups. However, at the first follow-up, a greater cortical thickness (mean 3.99 mm vs 2.89 mm), a higher grade of Bedi’s classification (49.2% of grade 3 vs 57.7% of grade 2), and a greater degree of color Doppler (44.6% of grade 2 vs 42.3% of grade 1) were identified in the non-infected group when compared to the previously infected group. Moreover, these statistically significant differences were also found in the second follow-up, with a larger number of lymph nodes (mean 6.6 vs 5.23), greater cortical thickness (mean 4.98 mm vs 3.49 mm), higher grade of Bedi’s classification (43.1% of grade 3 vs 38.5% of grade 2), and higher grade of color Doppler (53.8% of grade 2 vs 46.2% of grade 1) in the group who had not passed previously the infection. Regarding diameter values, no statistical differences were observed throughout the study (see Table 3 and Figs. 5 and 6).

In addition, the analysis of variance (ANOVA) test to assess disparity between the four radiologists showed a cortical thickness mean value in the second follow-up of 4.54 mm and 4.68 mm in US examinations performed by the expert radiologists (1 and 2) and of 4.36 mm and 4.98 mm in those made by third-year residents (radiologists 3 and 4) with non-statistically significant differences (*p* value = 0.81) (Table 4). Finally, a supplementary US follow-up examination was offered to 74 patients (81.3%) who presented a cortical thickness greater than 3 mm after the second dose. The follow-up was available in 43.3% of patients (32/74). Out of these 32 patients, 17 (53.1%) achieved normalization after 1 month, 6 (18.8%) after 2 months, 5 (15.6%) after 3 months, 1 (3.1%) after 4 months, and 3 (9.4%) continued presenting a cortical thickness greater than 3 mm the fourth month after the second vaccine dose administration. During this follow-up period, no one of the patients achieved basal cortical values.

**Table 4:** Total number of US examinations performed by each radiologist in the second follow-up and cortical thickness mean value for its explorations. Non-statistically significant differences in ANOVA test (*p* value > 0.05)

	Radiologist 1	Radiologist 2	Radiologist 3	Radiologist 4
US examinations 2nd follow-up (total number, %)	31 (34%)	15 (16.5%)	33 (36.3%)	12 (13.2%)
Cortex (mean, mm ± SD)	4.54 ± 1.77	4.68 ± 1.85	4.36 ± 2.23	4.98 ± 5.08
*p*	0.81			

## Discussion

There have been attempts to describe the effects of COVID-19 vaccination on locoregional lymph nodes using US examination, and small case studies of hyperplastic lymph nodes after injection of COVID-19 have been described in the literature [2, 5, 6]. However, a lack of detailed information reporting gradual imaging of axillary nodes in COVID-19 vaccinated patients exists. This is the first prospective study that examined nodal features before vaccination and over the dose injections of one type of mRNA vaccine, adding new insights about nodal morphologic dynamics and hyperplastic reaction in recipients.

Multiple online breast imaging forums have already reported current experience in terms of unilateral axillary lymphadenopathy growth in the setting of recent vaccination [2]. Influenza, measles, smallpox, anthrax, and Bacille Calmette-Guerin vaccines have all been implicated in occasional axillary lymphadenopathy [5], but prior to the implementation of COVID-19 vaccination, immunizations were considered a rare cause of benign reactive axillary lymphadenopathy. COVID-19 vaccines have associated a high immune response with greater percentages of patients developing hyperplasia of draining lymph nodes on the injected arm [6].

Pfizer-BioNTech belongs to a new generation of vaccines based on messenger RNA (mRNA), a genetic molecule used by our cells to produce proteins. The vaccine consists of a modified, non-replicating mRNA against a SARS-CoV-2 full-length spike protein, encapsulated in lipid nanoparticles. After dilution, the vaccine is administered intramuscularly in the deltoid muscle and nanoparticles of vaccine induce infiltration of neutrophils and antigen-presenting cells (monocytes and dendritic cells) into the injection site, as well as to the adjacent draining lymph nodes [7, 8]. These cells internalized vaccine contents, translate the mRNA, and, at the corresponding draining lymph nodes [7], present the protein to T-lymphocytes, which are “primed” to produce specific immune response against the virus [7–9].

In our study, all vaccinated volunteers included were health care workers from our center. Due to the higher proportion of female employees, the largest percentage of the sample was represented by women (79%) and the mean age was in the median age of the population (43.76), with a range between 20 and 65 years old. Out of 91 patients, 26 were previously infected with COVID-19, representing approximately 29% of the sample, a lesser percentage compared to the total number of infected health care workers in our country at this date (40%) [10].

According to our data, the most frequent number of visible nodes in patients without pathological conditions is approximately three, with a mean diameter and cortical thickness of 15.8 mm and 1.6 mm, respectively, categorized as grade 1 according to Bedi’s classification as well as at Doppler signal evaluation. Due to the absence of information about normal nodal imaging in US evaluation, these variables values could be included as standard lymph node sonography criteria for accurate assessment and to distinguish normal from pathological findings.

After the first injection, nodal changes were evidenced in all patients, except 1, who did not show induced hyperplasic lymphadenopathies, probably due to a recent history of SARS-COV2 infection. Statistical analysis of global variations showed an increase of 1.8 in the mean number of nodes, 2.1 mm in cortical thickness, and 5.3 mm in diameter. Regarding ordinal variables, 58 patients (64%) presented with suspicious nodes (types 3, 4, 5, and 6 in Bedi’s classification), and 60 patients (66%) showed an elevated Doppler signal (degrees 2 and 3). Additionally, a progressive increase of all variables was noted after the second dose injection with the greatest number of patients presenting suspicious nodes in Bedi’s classification (77 patients, 85%). Therefore, in line with previous studies, progressive ipsilateral nodal reaction appeared in practically all patients included in our study with a robust response after the second shot. Our results revealed that vaccine-induced hyperplastic axillary nodes showed benign characteristics more frequently, with mild cortical thickness and preserved uniformity, and classified mostly as Bedi type 3 in both follow-ups (43% and 42%, respectively). However, in oncologic follow-ups or patients recently diagnosed with breast cancer, malignancy exclusion is required [5], even in Bedi type 3, and percutaneous US-guided needle biopsy or a short-term follow-up ultrasound is necessary. Recommendations for follow-up vary widely, ranging from several weeks to months, so further investigations are needed to clarify data-driven guidelines.

Regarding our statistical control quality to analyze disparity between four radiologists, the variable registered was the maximum cortical thickness due to its easier comparative value and its higher influence in clinical management. Moreover, the second follow-up US examination was selected because of the cumulative effect of vaccination in nodal reaction with more evident changes in this last scan.

Additional analyses performed between patients who had previously passed the infection and patients who did not show a more significant axillary lymph node response to vaccination in patients who were not previously infected by SARS-CoV-2 even when both groups had shown the same nodal US characteristics at baseline examination. There could be several explanations for this seemingly paradoxical response. It would seem logical that spike protein presentation by antigen-presenting cells (APCs) has already occurred in other local sites in previously exposed patients during the infectious process, so that a second clonal expansion at a local site could be less relevant. Furthermore, studies of cell infiltration after mRNA vaccination show that the level of cell infiltration (neutrophils and APCs) at sites of injection seems to be at its highest 24 h after vaccination [7] so it is possible that cellular proliferation processes (which we equate with lymph node size) occur closer to the time of injection, as do systemic symptoms [8]. A more frequent, serial, follow-up of lymph node responses is necessary to confirm these results.

Regarding lymphadenopathy reaction outcome, more than 50% of patients normalized their cortex values the month after the second vaccination. More studies should be performed to determine the time required to reach basal cortical values.

Our study has several limitations, such as the small number of patients enrolled, the restriction of this research to one mRNA vaccine, and the absence of US examination of other nodal locations (i.e., subclavian, submandibular). Moreover, imaging examinations were obtained using two different US equipment and the selection of health care workers as the target population causes a sample selection bias, not having the possibility to report nodal features changes in extreme ages. Unfortunately, long-time follow-up data are not extensively available.

In conclusion, both doses of COVID-19 vaccine induced a significant increase of all axillary lymph node parameters, especially in coronavirus-naïve patients, adding new insights to previous observations of local adverse effects. To properly manage US axillary lymphadenopathy in the era of COVID19 vaccination, this study may further aid in identifying subgroups of patients that need close monitoring and perhaps for developing guidelines for clinical use. However, further research should be performed to understand and decipher underlying mechanisms.

## Abbreviations

2019-nCOV: New coronavirus disease
ANOVA: Analysis of variance
APC: Antigen-presenting cells
COVID-19: Coronavirus disease 2019
FDA: Food and Drug Administration
mRNA: Messenger ribonucleic acid
SARS-CoV2: Severe acute respiratory syndrome coronavirus
US: Ultrasound

## Declarations

### Guarantor

The scientific guarantor of this publication is Dr. Pina.

### Statistics and biometry

One of the authors has significant statistical expertise.

### Methodology

• prospective

• observational study

• performed at one institution
